# Metabolic Remodeling, Inflammasome Activation, and Pyroptosis in Macrophages Stimulated by *Porphyromonas gingivalis* and Its Outer Membrane Vesicles

**DOI:** 10.3389/fcimb.2017.00351

**Published:** 2017-08-04

**Authors:** Andrew J. Fleetwood, Man K.S. Lee, William Singleton, Adrian Achuthan, Ming-Chin Lee, Neil M. O'Brien-Simpson, Andrew D. Cook, Andrew J. Murphy, Stuart G. Dashper, Eric C. Reynolds, John A. Hamilton

**Affiliations:** ^1^Department of Medicine, University of Melbourne, Royal Melbourne Hospital Parkville, VIC, Australia; ^2^Haematopoiesis and Leukocyte Biology, Baker Heart and Diabetes Institute Melbourne, VIC, Australia; ^3^Oral Health Cooperative Research Centre, Melbourne Dental School, Bio21 Institute, University of Melbourne VIC, Australia

**Keywords:** macrophages, metabolism, inflammasome, *P. gingivalis*, vesicles, pyroptosis, cytokines and inflammation, glycolysis and oxidative phosphorylation

## Abstract

*Porphyromonas gingivalis* is one of the bacterial species most closely associated with periodontitis and can shed large numbers of outer membrane vesicles (OMVs), which are increasingly thought to play a significant role in bacterial virulence and pathogenicity. Macrophages are amongst the first immune cells to respond to bacteria and their products, so we sought to directly compare the response of macrophages to *P. gingivalis* or its purified OMVs. Macrophages stimulated with OMVs produced large amounts of TNFα, IL-12p70, IL-6, IL-10, IFNβ, and nitric oxide compared to cells infected with *P. gingivalis*, which produced very low levels of these mediators. Both *P. gingivalis* and OMVs induced a shift in macrophage metabolism from oxidative phosphorylation (OXPHOS) to glycolysis, which was supported by enhanced lactate release, decreased mitochondrial oxygen consumption with reduced spare respiratory capacity, as well as increased mitochondrial reactive oxygen species (ROS) production. Corresponding to this metabolic shift, gene expression analysis of macrophages infected with *P. gingivalis* or stimulated with OMVs revealed a broad transcriptional upregulation of genes critical to glycolysis and a downregulation of genes associated with the TCA cycle. Upon examination of inflammasome signaling and pyroptosis it was found that *P. gingivalis* did not activate the inflammasome in macrophages as the mature forms of caspase-1, IL-1β, and IL-18 were not detected and there was no extracellular release of lactate dehydrogenase (LDH) or 7-AAD staining. In comparison, macrophages stimulated with OMVs potently activated caspase-1, produced large amounts of IL-1β, IL-18, released LDH, and were positive for 7-AAD indicative of pyroptotic cell death. These data directly quantitate the distinct effects of *P. gingivalis* and its OMVs on macrophage inflammatory phenotype, mitochondrial function, inflammasome activation, and pyroptotic cell death that may have potential implications for their roles in chronic periodontitis.

## Introduction

*Porphyromonas gingivalis* is recognized as a keystone pathogen (Hajishengallis et al., 2012) and is one of the bacterial biofilm species isolated from subgingival plaque most strongly associated with clinical indicators of periodontitis, including increased pocket depth and bleeding on probing (Socransky et al., 1998; Komiya et al., 2000). A common feature of Gram-negative bacteria, like *P. gingivalis*, is the biogenesis of outer membrane vesicles (OMVs), which are spherical membrane structures that are increasingly thought to play a significant role in microbial virulence and not simply by-products of bacterial cell wall damage or lysis (Kuehn and Kesty, 2005; Ellis and Kuehn, 2010). *P. gingivalis* OMVs are enriched for the pathogen's major virulence factors such as gingipains (Arg- and Lys-specific proteolytic enzymes) and lipopolysaccharide (LPS) (Veith et al., 2014). Due to the small size of OMVs (50–70 nm in diameter) they spread more readily in tissues than their larger parent cells (Kuehn and Kesty, 2005; Darveau, 2010). As a result, *P. gingivalis* OMVs are highly immunogenic and have been found to induce infiltration of neutrophils in connective tissue (Srisatjaluk et al., 1999) and promote macrophage foam cell formation (Qi et al., 2003).

Recently, metabolic reprogramming in host immune cells, particularly in macrophages and dendritic cells has been implicated in regulating their phenotype and function (O'Neill and Pearce, 2016). Macrophages activated with LPS and IFNγ (so called M1 macrophages) shift their glucose metabolism from oxidative phosphorylation (OXPHOS) to glycolysis and this metabolic shift is central to their production of mediators associated with an M1 phenotype (e.g., NO) (Tannahill et al., 2013). Likewise the commitment of IL-4 stimulated macrophages (so called M2 macrophages) to OXPHOS to generate ATP is critical to their adoption of a M2 phenotype (Vats et al., 2006; Huang et al., 2014). A detailed comparison of metabolism in M1 vs. M2 macrophages identified specific metabolic pathways in both cell types that were critical in governing their polarization (Jha et al., 2015). Many recent studies have examined the links between glycolysis and cell effector function. For example, LPS-induced glycolysis enables dendritic cell maturation (Everts et al., 2014) whilst glycolysis is involved in inflammasome activation (Masters et al., 2010; Tannahill et al., 2013; Moon et al., 2015) and promotion of antibacterial responses in macrophages (Cordes et al., 2016; Lampropoulou et al., 2016). Much of this important information has been generated with purified LPS (reviewed in O'Neill et al., 2016) with relatively few studies (Garaude et al., 2016; Gleeson et al., 2016) addressing the impact of viable bacteria on cellular metabolism.

*P. gingivalis* has been shown to survive within macrophages (Wang et al., 2007; Wang and Hajishengallis, 2008; Slocum et al., 2014) and myeloid dendritic cells where it reprograms them to induce an immunosuppressive T cell effector response (Zeituni et al., 2009). Indeed, myeloid dendritic cells have been suggested to disseminate *P. gingivalis* from the oral mucosa to atherosclerotic plaques (Carrion et al., 2012). The ability of *P. gingivalis* to persist intracellularly is intriguing given the link between periodontal disease and certain systemic inflammatory conditions (Hajishengallis, 2015). Pyroptosis is a programmed form of proinflammatory cell death that allows the elimination of intracellular pathogens (Franchi et al., 2012; Aachoui et al., 2013). Pyroptosis occurs following activation of the cytosolic inflammasome signaling complex, which generates active caspase-1 leading to pore formation and the release of cytosolic contents (e.g., LDH) and production of the inflammatory cytokines IL-1β and IL-18 (Shi et al., 2015). There is conflicting evidence as to whether *P. gingivalis* can activate the inflammasome in macrophages, which in large part seems to be due to differences in cell populations studied (Taxman et al., 2006, 2012; Slocum et al., 2014). Another complication has been the gingipain-mediated degradation of the major readouts used to determine inflammasome activation and pyroptosis (Jung et al., 2015). *P. gingivalis* evasion of inflammasome activation would provide an intracellular niche for the pathogen to survive and disseminate to distant sites.

*P. gingivalis* OMVs can also persist within the lysosomes of host cells (Gui et al., 2016), are potent activators of Toll-like receptors (TLRs) (Cecil et al., 2016) and can deliver virulence factors deep into host tissues (Mashburn-Warren and Whiteley, 2006; Darveau, 2010). It is currently unknown whether *P. gingivalis* OMVs activate the inflammasome or induce pyroptotic cell death. The host response to OMVs is likely to be different to the parent bacterium as their phospholipid, gingipain and LPS profiles are distinct (Veith et al., 2014; Ho et al., 2015; Xie, 2015; Gui et al., 2016). It has been speculated that *P. gingivalis* OMVs act as a virulence factor secretion system that contributes to pathogenicity partly by supporting evasion of host immune defense mechanisms (Gui et al., 2016). In agreement with this a recent study found that the ability of *P. gingivalis* to subvert a local immune response was due to OMVs rendering monocytes unresponsive to live *P. gingivalis* (Waller et al., 2016). To better understand the immunostimulatory capabilities of OMVs, we recently developed a high-sensitivity flow cytometry method to enumerate *P. gingivalis*-derived OMVs (Cecil et al., 2016). This method allows removal of non-OMV associated material (e.g., fimbriae and secreted proteins; Nakao et al., 2014) to yield highly purified single lipid-bilayer vesicles. We found that the immunogenicity of vesicles purified from three periodontal pathogens (as measured by TLR activation) could be better differentiated when standardized using vesicle number compared to protein concentration (Cecil et al., 2016). To our knowledge there have been no studies directly comparing the immune responses elicited by *P. gingivalis* or its purified OMVs. By utilizing our protocol for enumerating vesicles (Cecil et al., 2016) we were able to directly compare the effect of *P. gingivalis* and OMVs at identical infection ratios on macrophage biology. Such studies may provide a more accurate understanding of their immunogenicity.

In the current study, we show that *P. gingivalis* and its OMVs induce a metabolic shift in macrophages from OXPHOS to glycolysis whilst OMVs potently activate proinflammatory cytokine production, inflammasome signaling, and pyroptotic cell death in macrophages. *P. gingivalis* only weakly activated macrophage cytokine production and did not activate the inflammasome or induce pyroptosis. These findings highlight the distinct ways in which *P. gingivalis* and its OMVs modulate the host immune response and underscore the potent stimulatory nature of OMVs, which may help explain their unique functions in periodontal disease.

## Materials and methods

### Macrophage culture

Murine BMM (from 8 to 12 week old C57BL/6 mice) and human MDM were prepared as before (Fleetwood et al., 2015). Briefly, bone marrow cells were isolated from femurs of mice and cultured in RPMI 1640 medium, supplemented with 10% heat-inactivated FCS, 2 mM GlutaMax-1, 100 U/ml penicillin, and 100 μg/ml streptomycin in the presence of M-CSF (2,000 U/ml). On day 4, non-adherent cells were collected and cultured for a further 3 days in M-CSF (2,000 U/ml) to derive BMM. Adherent BMM were harvested on day 7 at which stage they express the major macrophage surface markers (e.g., CSF-1R, F4/80, and Mac-1) (Lari et al., 2007). Human monocytes were purified from buffy coats (Red Cross Blood Bank, Melbourne, VIC, Australia), using RosetteSep Ab mixture (Stem Cell Technologies, Vancouver, BC, Canada), which negatively selects CD14^+^ monocytes, followed by Ficoll-Paque density gradient centrifugation (Way et al., 2009). Cells were then cultured in RPMI 1640 (supplemented as above) for 7 days in M-CSF (2,000 U/ml) to differentiate them into MDM (Lacey et al., 2012). All experiments were approved by the Royal Melbourne Hospital Research Foundation and Animal Ethics Committee and conducted in compliance with the guidelines of the National Health and Medical Research Council.

### Bacterial culture and enumeration

*P. gingivalis strain* W50 (ATCC 53978) was obtained from the culture collection of the Oral Health Cooperative Research Centre at the Melbourne Dental School. *P. gingivalis* W50 was grown and harvested as described (O'Brien-Simpson et al., 2000; Lam et al., 2016). Growth conditions of batch cultures were monitored at 650 nm using a spectrophotometer (model 295E, Perkin-Elmer, Germany). As before (Cecil et al., 2016), cells were harvested during late exponential growth by centrifugation (7,000 g, 20 min at 4°C) and enumerated (number/ml) by flow cytometry using a Cell Lab Quanta SC flow cytometer (Beckman Coulter, Australia) and a LIVE/DEAD BacLight™ Bacterial Viability Kit (Life Technologies, Australia). Bacteria were resuspended in 0.01 M phosphate buffered saline (PBS, Sigma-Aldrich), pH 7.4, before incubation with macrophages. Aliquots of *P. gingivalis* were heat-killed at 70°C for 1 h, as described (Palm et al., 2013).

### Isolation, purification, and enumeration of outer membrane vesicles

OMVs were purified (from W50 ATCC 53978) and enumerated as described in detail here (Cecil et al., 2016). Briefly, bacteria were grown to late exponential phase and whole bacterial cells were removed by centrifugation at 8,000 × g for 30 min at 4°C (using a F10BCI-6x500 g rotor installed in an Avanti J-25I Centrifuge, Beckman Coulter). The collected supernatant was filtered through a 0.22 μm filter (Merck-Millipore, Australia) and then concentrated through a 100-kDa filter using a tangential flow filtration Minimate TFF System (PALL Life Sciences, Australia). The collected concentrate was centrifuged at 100,000 × g for 2 h at 4°C (using a JA-30.50 Ti fixed angle rotor installed in an Avanti J-30I Centrifuge, Beckman Coulter) to yield a crude OMV preparation. Highly purified OMVs were prepared using OptiPrep™ density gradient centrifugation. Gradient fractions containing purified OMVs were identified using a Qubit 2.0 Fluorometer (Life Technologies). Fractions containing the purified OMVs were pooled and washed with 0.01 M PBS (Sigma-Aldrich), pH 7.4 at 150,000 × g for 2 h at 4°C (using a SW40 Ti rotor installed in an Optima L-80XP Ultracentrifuge, Beckman-Coulter) and resuspended in 0.22 μm filtered 0.01 M PBS. Aliquots of washed OMVs were enumerated (number/ml) using an Apogee A50-Micro Flow Cytometer calibrated for flow and event rates with Apogee Flow Systems Calibration Beads. Enumerated OMVs were resuspended in 0.01 M PBS (Sigma-Aldrich), pH 7.4, and the protein concentration quantitated by Qubit Protein Assay Kit (Life Technologies). Aliquots of OMVs were heat-inactivated at 70°C for 1 h, as described (Palm et al., 2013).

### Macrophage stimulation protocol

Macrophages were infected with viable *P. gingivalis*, heat-killed *P. gingivalis* (HK-*Pg*), OMVs or heat-inactivated OMVs (HI-OMVs) at a multiplicity of infection (MOI) of 10:1, 25:1 or 100:1 bacilli or OMV/cell for 2 h. After 2 h the extracellular bacteria/OMVs were removed and the macrophages were washed with PBS and then treated with fresh media (containing 100 U/ml penicillin, 100 μg/ml streptomycin to kill any remaining extracellular bacteria), and M-CSF (2,000 U/ml) for 24 h. This stimulation protocol was used throughout the study and based on our initial dose-response experiments (see Supplementary Figures 2A,B) and other studies (Taxman et al., 2012; Cecil et al., 2016; Waller et al., 2016) an MOI of 25:1 was chosen as the optimal dose for this study. For reference, OMVs (at an MOI of 25:1) was equal to a protein concentration of 3.0 μg/ml. In certain experiments cells were stimulated with *P. gingivalis*-derived LPS (Invivogen; LPS-PG Ultrapure at 100 ng/ml) for 24 h as indicated. These infection protocols were used throughout the study. In specific experiments the competitive gingipain inhibitors, KYT-1 and KYT-36 (Peptide Institute, Osaka, Japan) (Kadowaki et al., 2004) were included in the media (at 10 μM).

### PCR array and quantitative PCR

Total RNA was purified from BMM infected with *P. gingivalis* or OMVs using RNeasy Plus Mini kit (Qiagen, Valencia, CA) and was reverse transcribed using the RT^2^ First Strand cDNA kit (Qiagen). A RT^2^ Profiler PCR Array for Mouse Glucose Metabolism (PAMM-006, Qiagen) was performed according to the manufacturer's instructions (Thermal profile: Stage 1 95°C for 10 min, Stage 2 95°C for 15 s followed by 60°C for 1 min with 40 cycles) using an ABI PRISM 7900HT sequence detection system (Applied Biosystems, Carlsbad, CA). Genes on this array are grouped according to function as follows: TCA cycle, Glycolysis, Pentose Phosphate Pathway, Gluconeogenesis and Glucose Regulation, and Glycogen Metabolism. Data analysis was performed using the RT^2^ Profiler PCR Array Data Analysis Template v3.3 from Qiagen. A fold-change of 3, *p*-value < 0.05 was used as a cutoff. Data were normalized to five housekeeping genes—*Gusb, Hprt1, Hsp90ab1, Gapdh*, and *Actb*. For individual quantitative PCR analysis TaqMan probe/primer combinations for murine *Glut-1* (Mm00441480_m1), *Pfkfb3* (Mm00504650_m1), *Hk1* (Mm00439344_m1), and *Irg-1* (Mm01224532_m1) (Applied Biosystems) as before (Fleetwood et al., 2007). Threshold cycle numbers were transformed to ΔCt values, and results expressed relative to reference gene, HPRT. Assays were performed in triplicate and results expressed relative to HPRT from four independent experiments.

### ELISA, nitric oxide, LDH, and lactate quantification

IL-1β, IL-18, TNFα, IL-6, IL-12p70, IL-10 (Ready-Set-Go!; eBioscience), and IFNβ (PBL Assay Science) were quantified in the culture supernatants by ELISA according to manufactures instructions. NO production was measured by ${\text{NO}}_{2}^{-}$ quantification in a Griess reaction (Sigma). Extracellular LDH was detected with a Cytotoxicity Detection kit (Roche Diagnostics) and results are presented as the percentage of total intracellular LDH. Lactate concentration in supernatant was measured by colorimetric assay (Sigma) and read at 570 nM on a BioRad 680 Plate Reader. Five millimolars of 2-deoxyglucose (2DG; Sigma) was used as indicated.

### Metabolic assay

A Seahorse utility plate (Seahorse Bioscience) containing calibrant media (200 μl/well) together with Seahorse injector port and probe plate were incubated overnight in 37°C without CO_2_. The following day, media from Seahorse cell culture plate was replaced with Seahorse XF assay buffer (supplemented with 10 mM glucose and 2 mM glutamine) and incubated in CO_2_-free incubator at 37°C for at least half an hour. Macrophages that were infected (see stimulation protocol above) were then plated at 2 × 10^5^ cells/well in a 96-well Seahorse cell culture plate (Agilent XFe96) with one well per corner of the plate containing supplemented-media without cells, as background control. The designated injector ports were filled with 25 μl of the following MitoStress Test inhibitors; 1 μM Oligomycin (an ATP synthase inhibitor that allows calculation of mitochondrial O_2_ consumption); 1 μM Carbonyl cyanide-p-trifluoromethoxyphenylhydrazone [(FCCP), an ionophore that uncouples ATP synthesis from the electron transport chain to reveal maximal mitochondrial OXPHOS]; 1.5 μM Antimycin and 1.5 μM Rotenone (Complex I and III inhibitors that are used to reveal mitochondrial and non-mitochondrial O2 consumption) (Van den Bossche et al., 2015). The MitoStress Test Assay was run over 80 min on a Seahorse XF-e96 Bioanalyzer (Agilent) after calibration of utility plate with injector port plate as per manufacturer's instruction.

### Flow cytometry

As per manufacturer's instructions, stock solutions of MitoTracker Red CMXRos (1 mM), MitoSox Red (5 mM), and MitoTracker Green FM (1 mM) were made in high-quality, anhydrous dimethyl sulfoxide (DMSO) (Sigma). MitoSox Red was further diluted to a working solution of 5 μM in pre-warmed Hanks balanced salt solution (HBSS) with calcium and magnesium. Macrophages that were infected (see stimulation protocol above) were collected in ice-cold PBS and stained for 15 min at 37°C in a CO_2_ incubator with pre-warmed PBS (1% BSA) containing MitoTracker Red CMXRos (25 nM) or MitoTracker Green FM (25 nM) or with HBSS/Ca/Mg buffer containing MitoSox Red (5 μM). Following staining the cells were washed twice with fresh medium (at 37°C) and analyzed immediately to determine mitochondrial mass, membrane potential, and ROS production. In parallel cells were stained with 7-amino actinomycin D (7-AAD; R&D Systems) for 30 min at 4°C. 7-AAD is a membrane impermeant dye that is excluded from viable cells. After staining, cells were washed twice in pre-warmed PBS (1% BSA) and then analyzed immediately using a CyAn ADP Flow Cytometer (Beckman Coulter). As a positive control for 7-AAD staining, macrophages (uninfected) were treated with H_2_O_2_ for 60 min (1 mM; Sigma) prior to viability analysis (Fink and Cookson, 2006). For flow cytometric analysis, a typical forward and side-scatter gate was set to exclude dead cells and aggregates and a total of 10^4^ events in the gate were collected and analyses were performed using Kaluza v1.2 (Beckman Coulter) and data are expressed as mean fluorescence intensity (MFI). For 7-AAD staining the data are expressed as the percentage of positive cells for 7-AAD (± *SD*) compared to the untreated control.

### Immunoblots

Briefly, macrophages (3 × 10^6^) were lysed with RIPA buffer (25 mM Tris-HCl pH 7.6, 150 mM NaCl, 1% NP-40, 1% sodium deoxycholate, 0.1% SDS, and complete protease inhibitors) at 4°C and whole cell extracts collected. Lysates were clarified by centrifugation at 13,000 × g for 10 min at 4°C and protein concentrations were determined with a Bio-Rad protein assay kit and equal amounts of protein were separated on a NuPAGE Novex 4–12% Bis-Tris gel (Thermo Fisher Scientific) and transferred onto PVDF membrane (Bio-Rad). Membranes were probed with antibodies against caspase-1 (clone 14F468) (Novus Biologicals CO, USA), NLRP3 (D4D8T), Asc (D2W8U), IL-1β (AF-401-NA) (R&D Systems, MN, USA), β-actin (clone AC-74, Sigma-Aldrich, St. Louis, MO). Binding was visualized by incubation with horseradish peroxidase (HRP)-conjugated secondary antibodies and chemilluminescence (ECL). In particular experiments protein was precipitated from cell supernatants for detection of extracellular caspase-1, as described (Vanaja et al., 2016).

### Statistics analysis

ANOVA was used to analyse the statistical significance of the data. The Bonferroni *post-hoc* test was employed for the comparisons between groups (GraphPad Prism7). *p* < 0.05 indicate significance.

## Results

### Distinct cytokine and NO profiles induced by *P. gingivalis* and its OMVs

To compare cytokine production induced by the pathogen and its components, murine bone-marrow-derived macrophages (BMM) were infected with viable *P. gingivalis* or its purified OMVs for 2 h, after which the culture medium was removed and replaced with fresh medium containing antibiotics. To account for possible cytokine degradation by heat-labile proteases (e.g., gingipains) present on OMVs or its parent cell (Abe et al., 1998; Calkins et al., 1998; Stathopoulou et al., 2009), we also challenged cells with heat-killed *P. gingivalis* (HK-*Pg*) and HI-OMVs.

**Figure 1:**
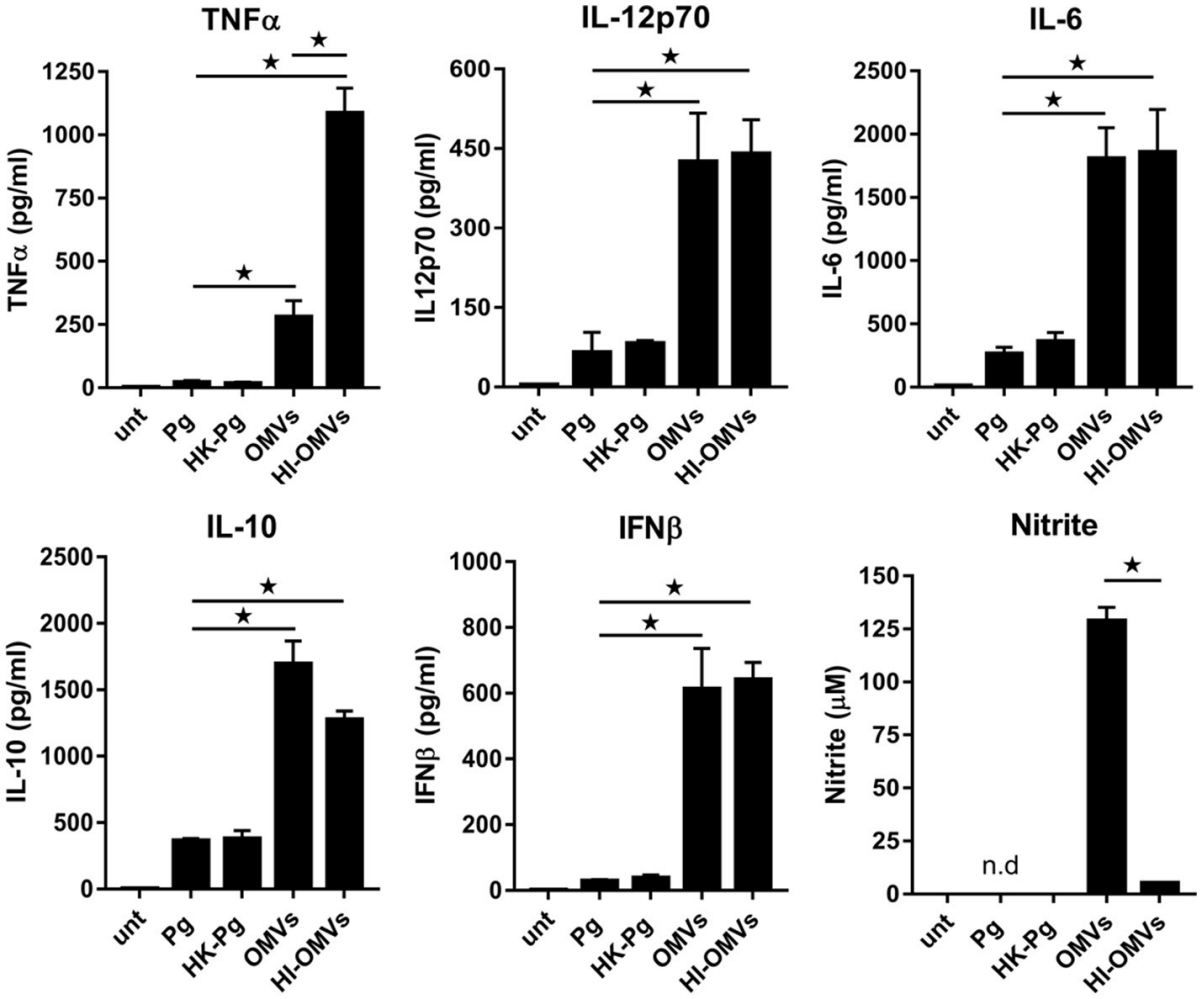
*P. gingivalis* and its OMVs differentially induce macrophage cytokine and nitric oxide release. BMM were infected (2 h at MOI of 25:1, see Materials and Methods) with viable *P. gingivalis* (*Pg*), heat-killed-*Pg* (HK-*Pg*), OMVs or heat-inactivated-OMVs (HI-OMVs), and release of TNFα, IL-12p70, IL-6, IL-10, IFNβ, and nitrite were measured at 24 h (6 h for TNFα). Cytokine levels (ELISA) and nitrite production (Griess reaction) were quantified and data are mean ± *SD* from four independent experiments (^*^p < 0.05; n.d, not detected).

BMM infected with *P. gingivalis* produced only modest amounts of TNFα, IL-12p70, IL-6, IL-10, and IFNβ compared to those stimulated with OMVs, which produced significantly (*p* < 0.05) higher levels of all of these mediators (Figure 1). BMM stimulated with OMVs produced large amounts of NO whilst cells infected with *P. gingivalis* failed to produce detectable NO (Figure 1). HK-*Pg* infection of BMM induced similar levels of TNFα, IL-12p70, IL-6, IL-10, and IFNβ compared to infection with viable *P. gingivalis*. Similar to *P. gingivalis*, HK-*Pg* did not induce detectable NO release from infected BMM. Of note, significantly (*p* < 0.05) higher levels of TNFα (Figure 1) were detected in the supernatants of BMM stimulated with HI-OMVs vs. OMVs, which is consistent with reports of gingipain-mediated degradation of this cytokine (Calkins et al., 1998). Interestingly, OMV-induced NO release from BMM was abolished when heat-inactivated (*p* < 0.05), suggesting a role for heat-labile protein(s) or enzyme(s) in promoting macrophage NO production. These data demonstrate the powerful immunostimulatory ability of OMVs by inducing large amounts of TNFα, IL-12p70, IL-6, IL-10, IFNβ, and NO from macrophages. In comparison, *P. gingivalis*-infected macrophages produce very low levels of these cytokines and failed to induce detectable NO. These findings are consistent with OMVs being enriched for virulence factors like LPS relative to their parent cells (Veith et al., 2014; Ho et al., 2015; Xie, 2015; Gui et al., 2016).

### *P. gingivalis* and its OMVs induce macrophage glycolysis

The studies of macrophage remodeling of their metabolism have mostly been done in response to purified *Escherichia coli* LPS (O'Neill et al., 2016). Thus, we wanted to investigate the impact of *P. gingivalis* and its purified OMVs on macrophage glucose metabolism. To investigate this, BMM and human monocyte-derived macrophages (MDM) were infected with viable *P. gingivalis* or purified OMVs (as above) and lactate release was measured as the end product of the glycolytic pathway.

**Figure 2:**
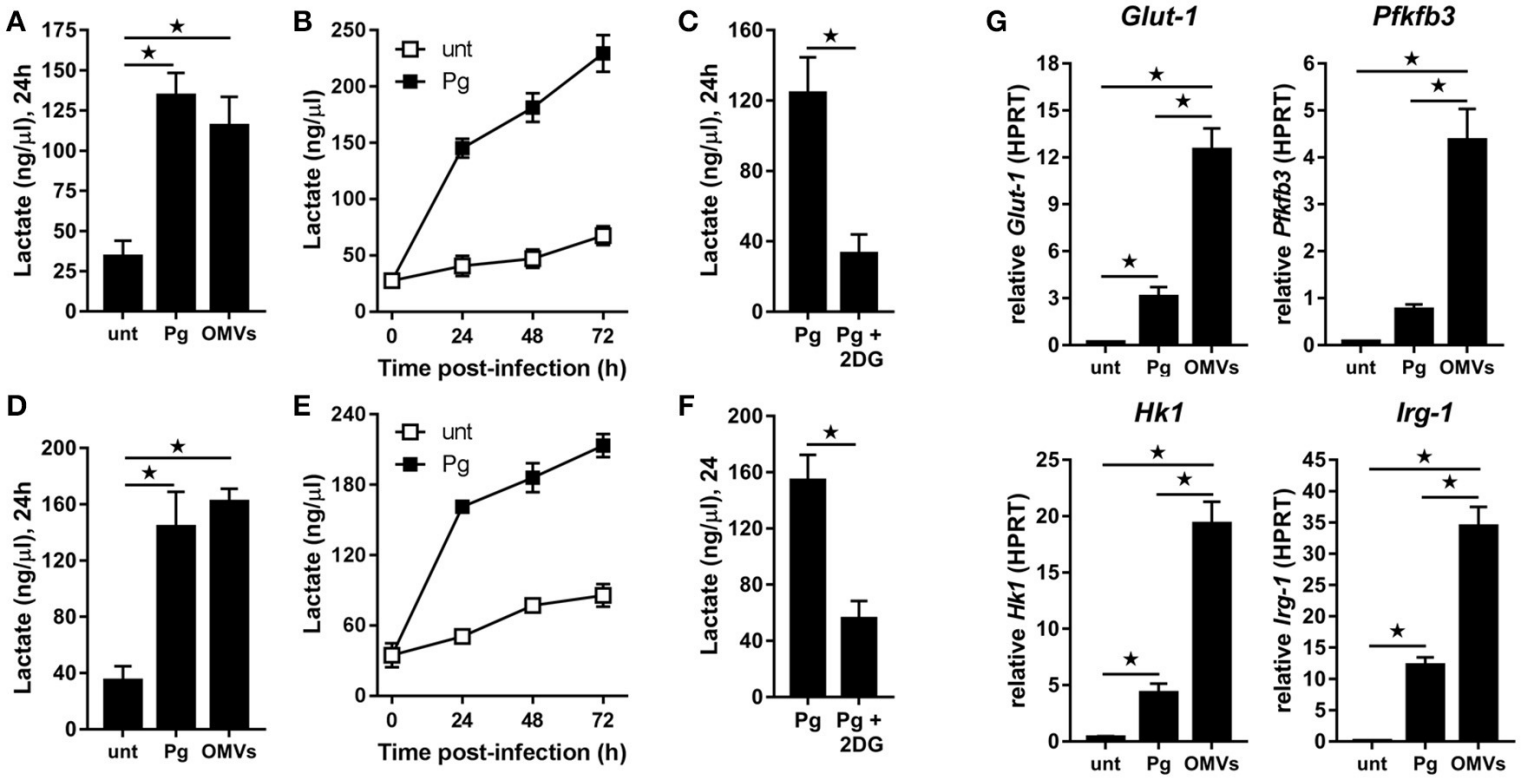
*P. gingivalis* and its OMVs induce glycolysis in macrophages. Secreted lactate concentrations from murine BMM **(A–C)** and human MDM **(D–F)** at indicated times (24–72 h) after infection with *P. gingivalis* (*Pg*) or its OMVs (25:1 MOI) (see Materials and Methods). Macrophages were treated with 2DG (5 mM) as indicated. **(G)** Gene expression by qPCR of *Glut-1, Pfkfb3, Hk-1*, and *Irg-1* were quantitated in BMM at 24 h (treated as in **A**). Data are mean ± *SD* from four independent experiments (^*^p < 0.05).

BMM (Figure 2A) and MDM (Figure 2D) demonstrated significantly (*p* < 0.05) increased lactate production at 24 h following infection with *P. gingivalis* or stimulation with OMVs relative to untreated cells. In the case of *P. gingivalis*-infected BMM (Figure 2B) and MDM (Figure 2E) the lactate levels increased over 72 h and could be blocked (*p* < 0.05) by pre-treatment of cells with the glycolytic inhibitor 2-deoxyglucose (2DG) (Figures 2C,F). HK-*Pg* and HI-OMVs also significantly (*p* < 0.05) increased lactate production from BMM (Supplementary Figure 1A) and MDM (Supplementary Figure 1D). We further found that *P. gingivalis*-derived LPS increased lactate production at 24 h in BMM and MDM relative to untreated cells (Supplementary Figures 1A,D). This shift toward glycolysis in BMM in response to *P. gingivalis* or OMVs was accompanied by an increased expression of *Glut-1, Pfkfb3*, and *Hk1* (Figure 2G), which encode proteins critically involved in the glycolytic pathway. Interestingly, we also found that BMM infected with *P. gingivalis* or stimulated with OMVs increased expression of *Irg-1* (Figure 2G), which has recently been postulated to impair OXPHOS in LPS-activated macrophages (Lampropoulou et al., 2016; Nemeth et al., 2016). Notably, BMM stimulated with OMVs had significantly (*p* < 0.05) higher expression of *Glut-1, Pfkfb3, Hk1*, and *Irg-1* relative to *P. gingivalis*-infected BMM. These data show for the first time that *P. gingivalis* (and its major components, OMVs and LPS) induce glycolysis in murine and human macrophages.

### *P. gingivalis* and its OMVs disrupt macrophage mitochondrial respiration

**Figure 3:**
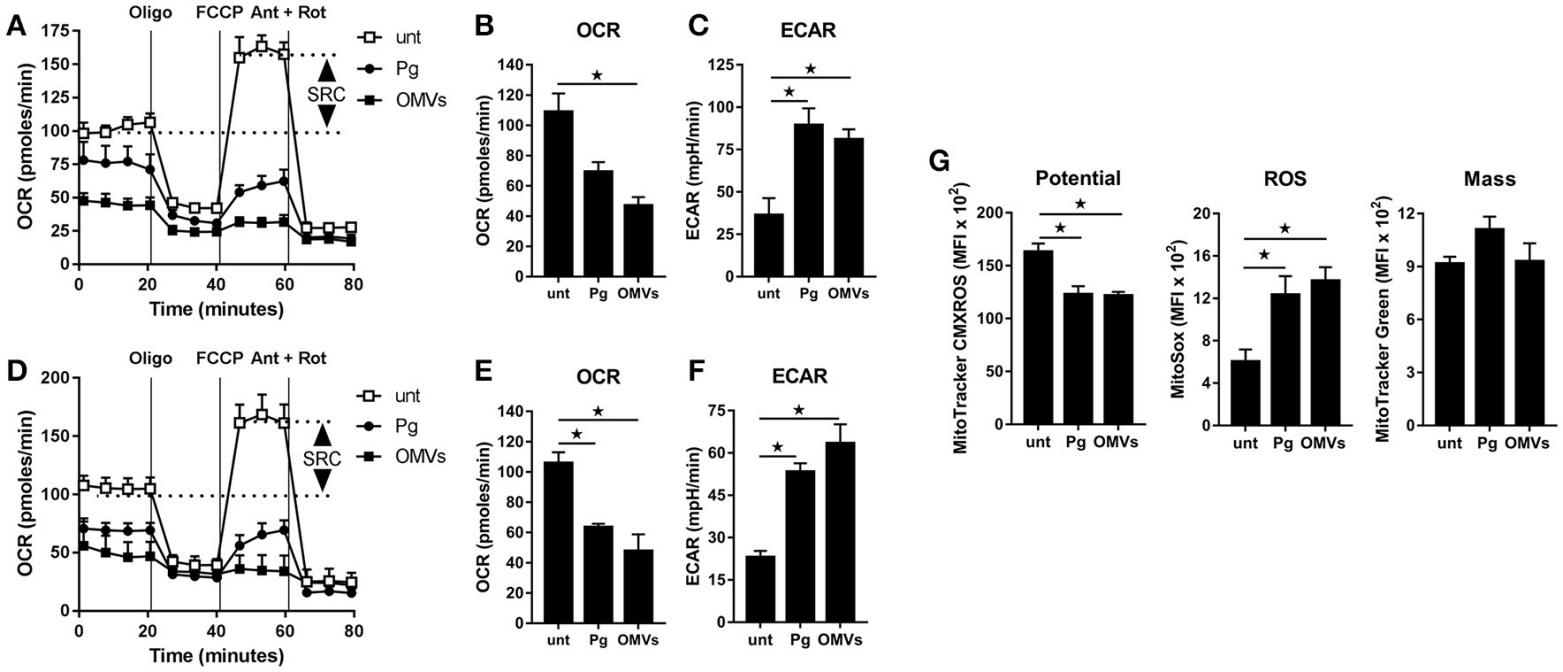
*P. gingivalis* and its OMVs disrupt mitochondrial respiration in macrophages. The mitochondrial respiration of BMM **(A–C)** and MDM **(D–F)** following infection with *P. gingivalis* (*Pg*) or OMVs (2 h at MOI of 25:1, see Materials and Methods) measured in a Seahorse XF-24 analyzer after 24 h. **(A,D)** Real-time oxygen consumption (OCR) was determined during sequential treatments with oligomycin (ATP-synthase inhibitor), FCCP, and antimycin-A + rotenone (ETC inhibitors) over 80 min. Spare respiratory capacity (SRC), the difference between maximal OCR, and the basal OCR, is depicted in the plots. Data represent means ± *SD* of triplicates. One of three experiments is shown. Basal OCR **(B,E)** and extracellular acidification rate (ECAR) **(C,F)** were analyzed (at 24 h) as readouts for oxidative phosphorylation (OXPHOS) and glycolysis, respectively. Data are mean ± *SD* from three independent experiments. **(G)** Mitochondrial membrane potential, ROS production and mass were determined at 24 h in BMM (treated as in **A**) by flow cytometry after staining with MitoTracker CMXROS, MitoSOX Red and MitoTracker Green FM, respectively. Data are mean fluorescence intensity (MFI) ± *SD* from three independent experiments (^*^p < 0.05).

To determine whether the observed increase in glycolysis was paralleled with changes to mitochondrial respiration we next performed extracellular flux analysis to measure the mitochondrial activity of untreated macrophages vs. those that were infected (as above) with *P. gingivalis* or stimulated with OMVs. As detailed in Figures 3A–C (for BMM) and Figures 3D–F (for MDM), changes in the oxygen consumption rate (OCR) and extracellular acidification rate (ECAR) were measured in response to oligomycin (oligo), FCCP, and Antimycin + Rotenone (Ant + Rot) injection (Everts et al., 2012). These analyses revealed BMM and MDM infected with *P. gingivalis* or stimulated with OMVs had a markedly diminished spare respiratory capacity (SRC), as indicated by the difference between maximal OCR (after FCCP injection) and the basal OCR (Figures 3A,D), consistent with a reduced commitment to OXPHOS in these cells (Huang et al., 2016). Basal OCR was significantly (*p* < 0.05) reduced in MDM that were infected with *P. gingivalis* or stimulated with OMVs (Figure 3E) whilst basal OCR was significantly (*p* < 0.05) reduced in OMV-stimulated BMM (Figure 3B). *P. gingivalis*-infected BMM had a reduced OCR but this did not reach statistical significance (Figure 3B). HK-*Pg*, HI-OMVs, and *P. gingivalis*-derived LPS also reduced the SRC of BMM and MDM (Supplementary Figures 1B,E) whilst HI-OMVs and LPS also significantly reduced their basal OCR (Supplementary Figures 1C,F). Consistent with their increased lactate generation (Figures 2A,D), BMM and MDM infected with *P. gingivalis* or stimulated with OMVs has a significantly (*p* < 0.05) increased ECAR (Figures 3C,F). Upon further examination of their mitochondrial function, we observed that *P. gingivalis*-infected and OMV-stimulated BMM had decreased mitochondrial membrane potential, increased mitochondrial ROS release with no significant change in mitochondrial mass (Figure 3G). These data show for the first time that murine and human macrophages switch their glucose metabolism from OXPHOS to glycolysis and generate mitochondrial ROS in response to *P. gingivalis* (and its major components, OMVs and LPS).

### *P. gingivalis* and its OMVs regulate macrophage metabolic gene expression

**Figure 4:**
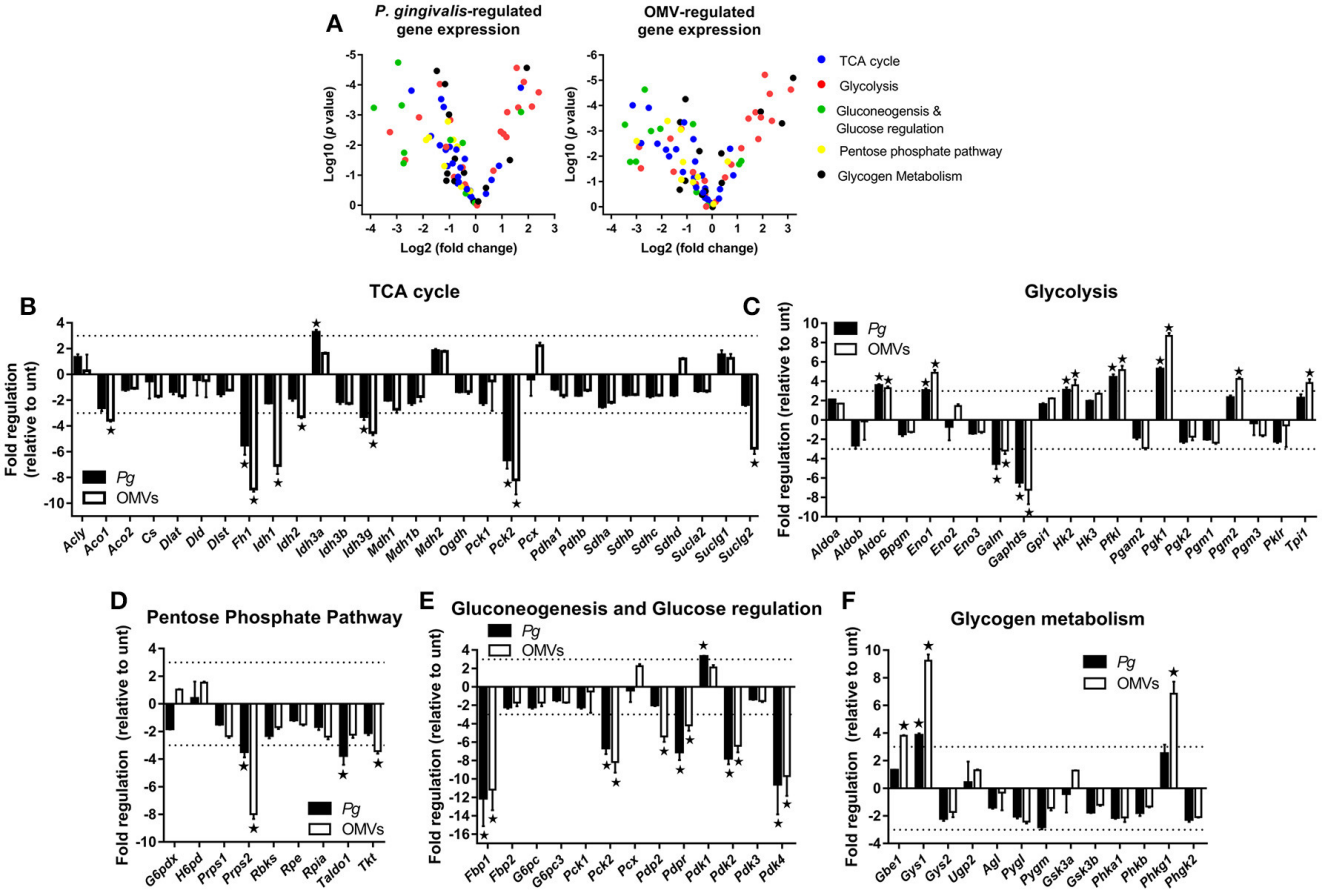
*P. gingivalis* and its OMVs regulate macrophage metabolic gene expression. **(A)** Volcano plots showing genes differentially expressed in BMM following infection with *P. gingivalis* or OMVs (25:1 MOI, see Materials and Methods) relative to untreated BMM after 24 h using a Glucose Metabolism PCR Array (PAMM-006Z). RNA was purified for cDNA synthesis and qPCR performed and **(B–F)** genes are grouped according to function and expression is shown relative to untreated BMM. Data are mean ± *SD* from three independent experiments. Genes with greater than three-fold difference from untreated cells (indicated with dotted line) were considered significant (^*^p < 0.05).

Our earlier data (see Figure 2G) revealed that *P. gingivalis* and its OMVs induced the expression of several key genes involved in glucose metabolism. As such, a qPCR array focused on glucose metabolism was used to measure changes in gene expression in *P. gingivalis* or OMV stimulated macrophages. BMM were infected (as above) with *P. gingivalis* or OMVs and changes in expression (of a panel of 84 genes) were compared to untreated cells at 24 h. The gene expression data relative to untreated cells for *P. gingivalis* and OMV treated BMM are summarized in Volcano plots (Figure 4A) and are grouped according to gene function (Figures 4B–F). Genes with greater than three-fold difference from untreated cells were considered significant.

From the panel of 84 genes that were analyzed, 20 genes were significantly (*p* < 0.05) regulated in BMM infected with *P. gingivalis* relative to untreated cells whilst 27 genes were significantly (*p* < 0.05) regulated in OMV treated BMM relative to control cells. In broad terms both *P. gingivalis* and OMVs induced similar expression profiles in BMM compared to control cells, with both stimuli reducing the expression of several TCA cycle genes (Figure 4B) whilst enhancing the expression of many glycolytic genes (Figure 4C). Additionally, *P. gingivalis* and OMV stimulation of BMM led to reduced expression of transcripts involved in the pentose phosphate pathway (Figure 4D) and in gluconeogenesis and glucose regulation (Figure 4E). OMVs tended to be a more potent stimulus at least under the current experimental conditions than *P. gingivalis*. Specific alterations in gene expression are discussed in more detail below.

*P. gingivalis* and OMVs significantly (*p* < 0.05) downregulated BMM expression of many genes involved in the TCA cycle like fumarase-1 (*Fh1)*, isocitrate dehydrogenase 3 gamma *(Idh3g)*, and phosphoenolpyruvate carboxykinase 2 (*Pck2)* (Figure 4B). Three key enzymes of the TCA cycle involved in the reactions that convert citrate to succinate, aconitase 1 *(Aco1)*, isocitrate dehydrogenase 1 and 2 *(Idh1/2)*, and succinyl-CoA ligase (GDP-forming) subunit beta *(Suclg2)* were specifically downregulated (*p* < 0.05) in response to OMVs (Figure 4B). On the other hand, expression of genes related to glycolysis like aldolase C *(Aldoc)*, enolase 1 *(Eno1)*, hexokinase 2 *(Hk2)*, phosphofructokinase *(Pfk1)*, and phosphoglycerate kinase 1 *(Pgk1)* were all enhanced (*p* < 0.05) in BMM following *P. gingivalis* or OMV stimulation (Figure 4C). Not all glycolytic genes were upregulated with aldose 1-epimerase *(Galm)* and glyceraldehyde-3-phosphate dehydrogenase, spermatogenic *(Gapdhs)* both being downregulated (*p* < 0.05) in BMM by *P. gingivalis* and OMVs (Figure 4C).

Macrophages that have committed to glycolysis following activation (e.g., via LPS) have increased flux through the pentose phosphate pathway (Krawczyk et al., 2010; Freemerman et al., 2014). Interestingly, we found that BMM infected with *P. gingivalis* or stimulated with OMVs tended to downregulate genes in this pathway (Figure 4D). Expression of phosphoribosyl pyrophosphate synthetase 2 (*Prps2*), transaldolase *1* (*Taldo1*), and transketolase (*Tkt*) were all reduced in BMM infected with *P. gingivalis* (*Prps2* and *Taldo1*) or stimulated with OMVs (*Prps2* and *Tkt*) (Figure 4D, *p* < 0.05). Genes involved in gluconeogenesis, namely fructose bisphosphatase (*Fbp1*) and phosphoenolpyruvate carboxykinase 2 (*Pck2*) were significantly (*p* < 0.05) downregulated in BMM treated with *P. gingivalis* or OMVs (Figure 4E) as were pyruvate dehydrogenase phosphatase regulatory subunit (*Pdpr*) and pyruvate dehydrogenase kinase isoforms 2/4 (*Pdk2/4*), which are regulators of glucose metabolism (Figure 4E). Glycogen branching enzyme (*Gbe1*), glycogen synthase 1 (*Gys1*), and phosphorylase b kinase gamma catalytic chain (*Phkg1*) were all enhanced (*p* < 0.05) in OMV stimulated BMM (Figure 4F).

Overall these data reveal that *P. gingivalis* and its OMVs reprogram the metabolic gene expression profile of macrophages by enhancing expression of genes involved in the glycolytic pathway whilst simultaneously decreasing expression of genes involved in the TCA cycle, pentose phosphate pathway, and the gluconeogenesis and glucose regulation pathways.

### Distinct inflammasome signaling and pyroptosis induced by *P. gingivalis* and its OMVs

**Figure 5:**
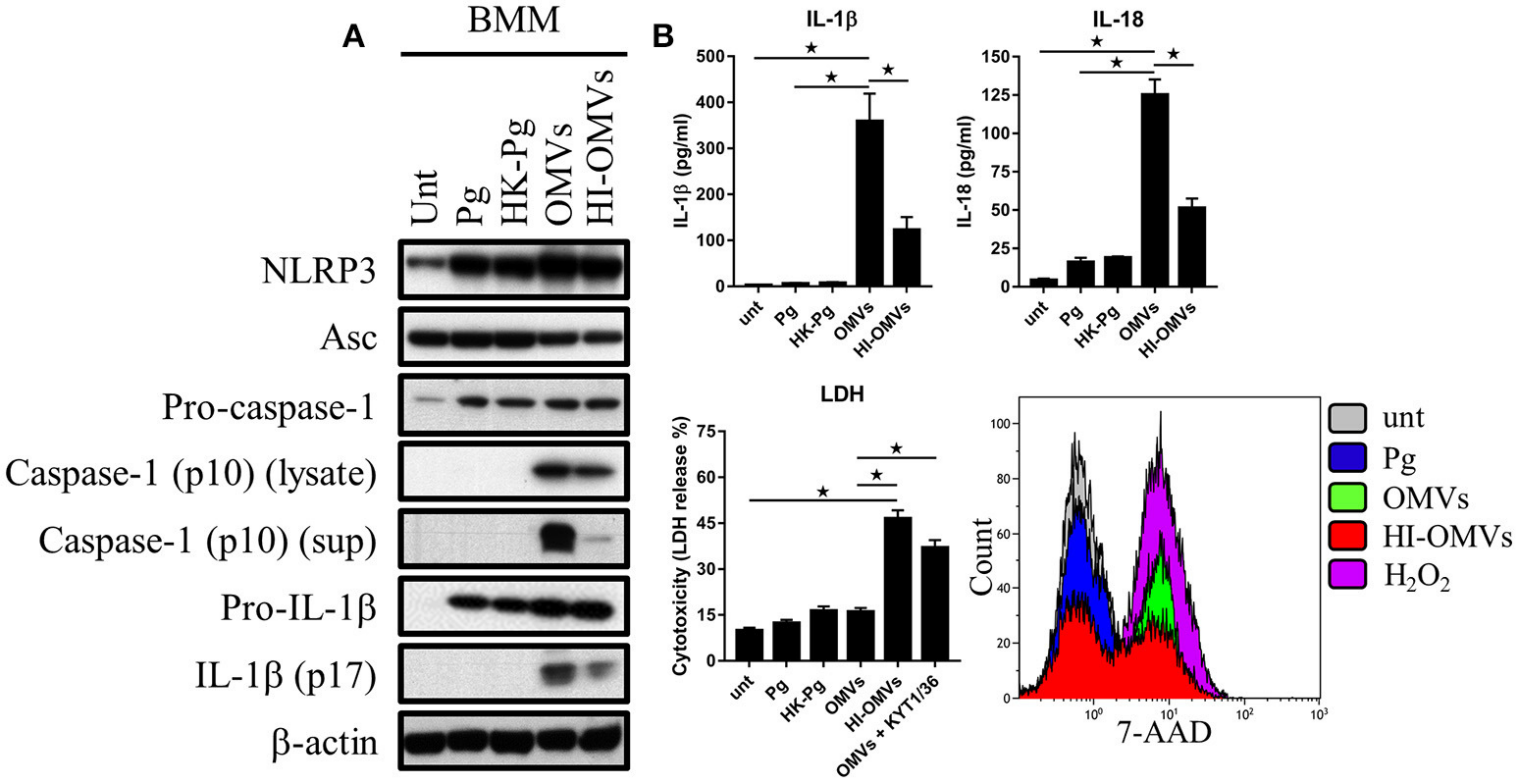
*P. gingivalis* and its OMVs differentially induce inflammasome signaling and pyroptosis in murine macrophages. BMM were infected as before (2 h at MOI of 25:1, see Materials and Methods) with viable *P. gingivalis* (*Pg*), heat-killed-*Pg* (HK-*Pg*), OMVs, or heat-inactivated-OMVs (HI-OMVs) and **(A)** the activation of inflammasome components in the lysates [or supernatants (sup) where indicated] measured after 24 h by Western blot; β-actin serves as a loading control throughout. Western blot data are representative of at least three independent experiments. **(B)** Production of IL-1β and IL-18 (by ELISA) from BMM was measured in the supernatant at 24 h. Data are mean ± *SD* from four independent experiments. Cell viability was determined by measuring extracellular LDH release and by 7-AAD exclusion (by flow-cytometry) at 24 h. 7-AAD is a membrane impermeant dye that is excluded from viable cells. The percent of 7-AAD positive (±*SD*) cells from three independent experiments was calculated relative to the untreated control. BMM treated with H_2_O_2_ (1 mM for 60 min) were included as positive control for 7-AAD. A representative histogram is shown. Cells were also treated where indicated with OMVs in the presence of KYT-1 and KYT-36 (10 μM of each). ^*^p < 0.05.

**Figure 6:**
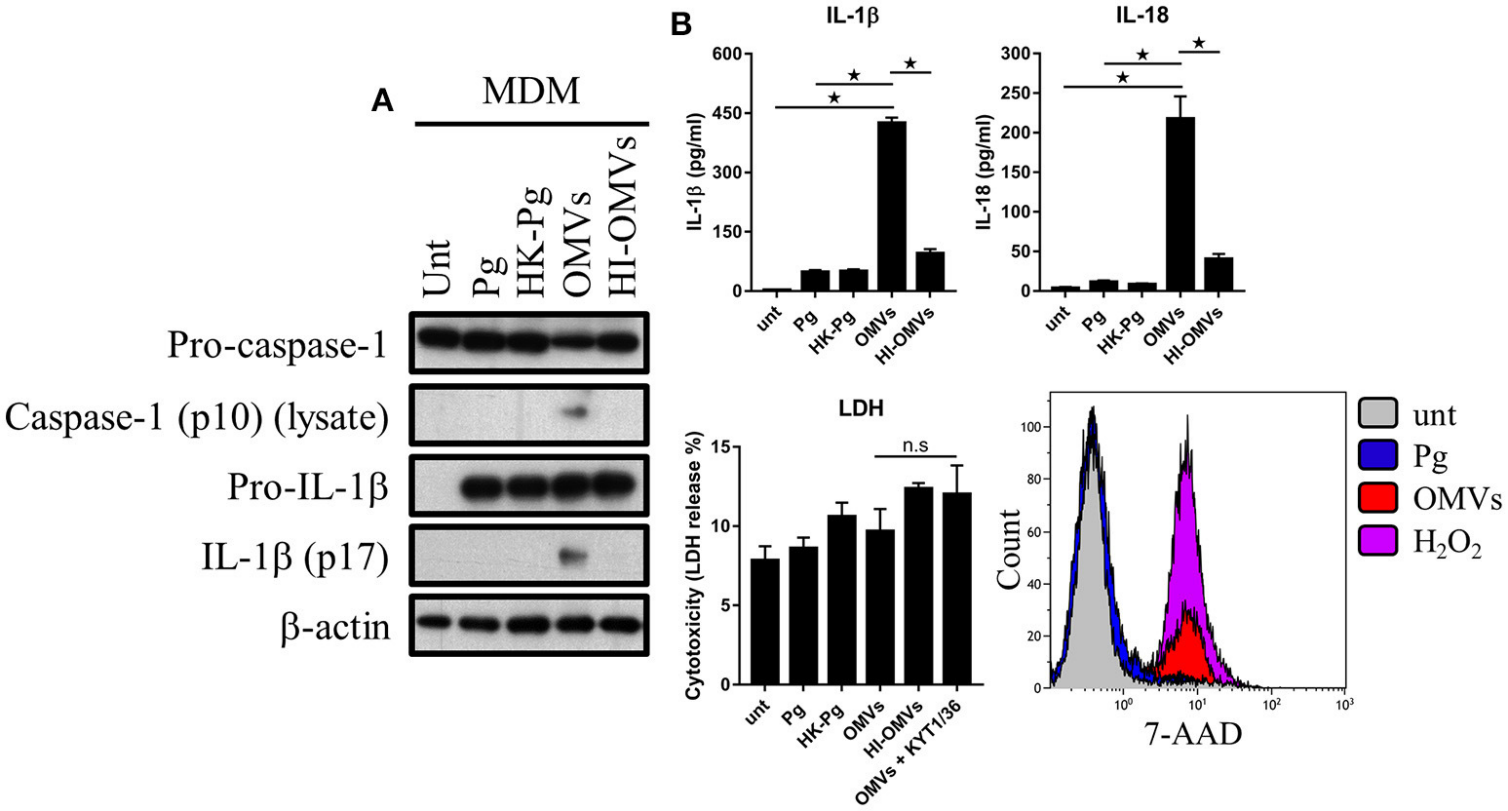
*P. gingivalis* and its OMVs differentially induce inflammasome signaling and pyroptosis in human macrophages. MDM were infected as before (2 h at MOI of 25:1, see Materials and Methods) with viable *P. gingivalis* (*Pg*), heat-killed-*Pg* (HK-*Pg*), OMVs, or heat-inactivated-OMVs (HI-OMVs) and **(A)** the activation of inflammasome components in the lysates was measured after 24 h by Western blot; β-actin serves as a loading control throughout. Western blot data are representative of at least three independent experiments. **(B)** Production of IL-1β and IL-18 (by ELISA) from MDM was measured in the supernatant at 24 h. Data are mean ± *SD* from four independent experiments. Cell viability was determined by measuring extracellular LDH release and by 7-AAD exclusion (by flow-cytometry) at 24 h. 7-AAD is a membrane impermeant dye that is excluded from viable cells. The percent of 7-AAD positive (±*SD*) cells from three independent experiments was calculated relative to the untreated control. MDM treated with H_2_O_2_ (1 mM for 60 min) were included as positive control for 7-AAD. A representative histogram is shown. Cells were also treated where indicated with OMVs in the presence of KYT-1 and KYT-36 (10 μM of each). ^*^p < 0.05: n.s, not significant.

Given that *P. gingivalis* and OMVs dramatically altered macrophage glucose metabolism and that cellular glucose metabolism is thought to regulate inflammasome activation (Everts and Pearce, 2014; O'Neill and Pearce, 2016), we next sought to address the effect of *P. gingivalis* and OMVs on macrophage inflammasome activation. In addition, we determined whether *P. gingivalis* and OMVs induced pyroptosis, which is a proinflammatory and lytic form of cell death (Schroder and Tschopp, 2010), that can occur following prolonged activation of the inflammasome (Miao et al., 2011). To investigate this BMM (Figure 5) and MDM (Figure 6) were infected with *P. gingivalis*, HK-*Pg*, OMVs, or HI-OMVs (as above) and inflammasome activation and pyroptosis measured at 24 h.

Treatment of BMM with *P. gingivalis*, HK-*Pg*, OMVs, or HI-OMVs induced the expression on the major inflammasome components NLRP3, pro-caspase-1, and pro-IL-1β. Asc was endogenously expressed and its levels were unchanged regardless of treatment (Figure 5A). Consistent with activation of the inflammasome, BMM treated with OMVs, and to a lesser extent HI-OMVs, triggered cleavage of pro-caspase-1 and pro-IL-1β into mature caspase-1 p10 (detected in the lysate and supernatant) and IL-1βp17 (Figure 5A), as well as inducing IL-1β and IL-18 secretion (Figure 5B). Moreover, OMV treatment of BMM led to the release of large amounts of IL-1β and IL-18 in an MOI-dependent manner (Supplementary Figure 2A) whereas infection with *P. gingivalis* only led to minimal amounts of these mediators being produced. A feature of pyroptosis is the formation of pores in the plasma membrane that allow the release of intracellular contents (Jorgensen and Miao, 2015). Consistent with the induction of pyroptosis, OMVs triggered significant release of the intracellular protein LDH from BMM (Figure 5B), which was only evident when BMM were treated with OMVs in the presence of the gingipain proteolytic inhibitors KYT-1 and KYT-36, or when stimulated with HI-OMVs (Figure 5B). These findings indicate that gingipains, which are enriched on OMVs (Veith et al., 2014), degrade extracellular LDH to mask this specific readout of pyroptosis. These findings concur with an earlier report of gingipain-mediated LDH proteolysis (Jung et al., 2015). As mentioned above, pyroptosis leads to the formation of pores in the plasma membrane that allow a membrane impermeant dye like 7-AAD to enter pyroptotic cells but not apoptotic or viable cells (Miao et al., 2011). We found that a significant (*p* < 0.05) percentage of BMM treated with OMVs (69% ± 8) and to a lesser extent HI-OMVs (46% ± 5) were positive for 7-AAD relative to untreated (7% ± 3) or *P. gingivalis* infected BMM (13% ± 5) (Figure 5B). BMM treated with OMVs at an MOI of 100:1 were nearly uniformly positive for 7-AAD (89% ± 7) (Supplementary Figure 2A). In summary, *P. gingivalis* did not trigger caspase-1 and IL-1β maturation, IL-1β, and IL-18 secretion or pyroptosis in BMM (Figure 5B). Thus, unlike their parent bacterium, OMVs trigger inflammasome activation and pyroptosis in murine macrophages.

We next addressed whether these observations were seen in MDM and found that OMVs, but interestingly not HI-OMVs, triggered caspase-1 and IL-1β maturation (Figure 6A) and IL-1β and IL-18 secretion (Figure 6B). Similar to BMM, OMVs potently induced IL-1β and IL-18 release from MDM in a MOI-dependent manner compared to *P. gingivalis* (Supplementary Figure 2B). Interestingly, despite clear inflammasome activation, there was no observable increase in LDH release indicative of pyroptosis from MDM in response to OMVs or OMVs + KYT-1/KYT-36 (Figure 6B). MDM are known to have diminished cell death responses, as measured by LDH release, relative to murine BMM (Bezbradica et al., 2017). A significant (*p* < 0.05) percentage of MDM treated with OMVs (23% ± 3) were positive for 7-AAD compared to untreated (4% ± 3) and *P. gingivalis* (6% ± 2) infected cells (Figure 6B). MDM treated with OMVs at an MOI of 100:1 were almost all positive for 7-AAD (84% ± 11) (Supplementary Figure 2B). Similar to BMM, MDM infected with *P. gingivalis* did not trigger caspase-1 and IL-1β maturation, IL-1β, and IL-18 secretion or pyroptosis. Combined these data show that *P. gingivalis* failed to activate the inflammasome in murine and human macrophages whereas purified OMVs provide the necessary priming and triggering signals to activate the inflammasome complex in both these populations. The ability of OMVs to activate the inflammasome (i.e., induce the mature forms of caspase-1 and IL-1β) appeared to be partly dependent on their expression of heat-labile protein(s) or enzyme(s), as these responses were impaired in BMM treated with HI-OMVs. In MDM, OMV-induced inflammasome activation was completely abolished when they were heat-inactivated. OMV enrichment for the gingipains relative to their parent cell (Veith et al., 2014) may partly explain the disparate macrophage inflammasome and pyroptotic responses to *P. gingivalis* compared to OMVs.

## Discussion

In this study we demonstrate that *P. gingivalis*, its OMVs and LPS, disrupt macrophage mitochondrial respiration and function and shift cellular metabolism toward glycolysis. We also provide important insights into the distinct responses of macrophages to OMVs compared to the parent bacterium. Macrophages stimulated with OMVs produce large amounts of inflammatory mediators and activate the inflammasome and pyroptotic cell death pathways. On the other hand, macrophages infected with *P. gingivalis* produce low levels of inflammatory cytokine and fail to activate the inflammasome or induce pyroptosis. These data help to further define the unique role of OMVs as the pathogen's major virulence factor and activator of host inflammation.

*P. gingivalis* OMVs are relatively small and stable and can penetrate host tissues where they activate an inflammatory host response (O'Brien-Simpson et al., 2009). However, quantitating their number in periodontitis is extremely difficult. In a previous study (Cecil et al., 2016), we found that for *P. gingivalis* cultures (at late exponential growth phase) the ratio of cells to OMVs was ~1:2,000. The levels of *P. gingivalis* in subgingival plaque are associated with disease severity and a threshold of around 10^6^*P. gingivalis* cells per site is required for disease progression (Gmur et al., 1989; Jervoe-Storm et al., 2007; Byrne et al., 2009). If we extrapolate (using the 1:2,000 ratio of parent cells to OMVs) this equates to ~2 × 10^9^ OMVs/site, which is well above the highest levels we use in the current study. Our data here do strongly support the notion that OMVs are potent activators of the host response (Ellis and Kuehn, 2010). We found macrophages produced large amounts of TNFα, IL-12p70, IL-6, IL-10, NO, and IFNβ in response to OMVs. Interestingly, IFNβ can act in an autocrine manner to promote IL-12p70, IL-10, and NO production in macrophages (Fleetwood et al., 2009), suggesting that IFNβ may be a central regulator of the macrophage cytokine response to OMVs. Consistent with this notion, macrophages infected with *P. gingivalis* produced comparatively low levels of IFNβ and were capable of only low level formation of IL-12p70 and IL-10 and failed to induce detectable NO. A recent study demonstrated a key role for IFNβ in *P. gingivalis*-induced periodontal disease (Mizraji et al., 2017). These data fit with the concept that a major role of OMVs is to activate host immune and inflammatory pathways to provide the nutrients, in the form of tissue breakdown products, that are necessary for the survival of the parent *P. gingivalis* cell (Hajishengallis, 2011, 2014).

There is a growing appreciation of the interdependency of metabolism and macrophage cell function (O'Neill et al., 2016). Much of this information has been generated via analysis of the metabolic reprogramming that occurs in LPS-stimulated macrophages with only a few studies using viable bacteria (Garaude et al., 2016; Gleeson et al., 2016). We demonstrate here that viable *P. gingivalis* induces a metabolic shift toward glycolysis in macrophages with attendant reduction in OXPHOS and mitochondrial function, as well as reduction in the expression of many key genes involved in the TCA cycle. A similar switch was observed in human and mouse macrophages stimulated with *P. gingivalis* OMVs and *P. gingivalis*-derived LPS. It might be expected then from these data that macrophages interacting locally with viable *P. gingivalis* or, for example, in the tissue with secreted OMVs (Mashburn-Warren and Whiteley, 2006; Darveau, 2010), will undergo metabolic reprogramming toward glycolysis. Interestingly, the disruption of OXPHOS and switch to glycolysis drives macrophage inflammatory responses (Cramer et al., 2003; Tannahill et al., 2013; Van den Bossche et al., 2015), and once the switch to glycolysis has been made these activated macrophages cannot be “repolarized” to adopt an anti-inflammatory phenotype (Van den Bossche et al., 2016). Activated macrophages are typically thought to utilize glycolysis for rapid ATP generation and clearance of intracellular pathogens by NO and ROS (O'Neill and Pearce, 2016; Van den Bossche et al., 2016). Here we found that *P. gingivalis* promoted glycolysis in macrophages without the attendant generation of NO, which in dendritic cells is required for the switch to glycolysis to occur (Everts et al., 2012; Everts and Pearce, 2014). In contrast, we found OMV induction of glycolysis was associated with high levels of NO production. Our data are consistent with reports of *P. gingivalis* inhibition (Wang et al., 2010) and OMV promotion (Imayoshi et al., 2011) of macrophage iNOS/NO generation. Unlike in dendritic cells, the switch to glycolysis in macrophages is not thought to be due to NO inhibition of OXPHOS (Everts et al., 2012; Everts and Pearce, 2014), but likely due to itaconate generation (Cordes et al., 2016; Lampropoulou et al., 2016). Itaconate is the product of an enzyme encoded by the immune responsive gene-1 (*Irg-1*) that converts cis-aconitate (derived from citrate) to itaconic acid (O'Neill and Pearce, 2016). Itaconate production is hugely increased in glycolytic macrophages where it functions as a central regulator of TCA cycle remodeling, via inhibition of succinate dehydrogenase (SDH) activity (Lampropoulou et al., 2016). In agreement with these studies the macrophage switch to glycolysis following *P. gingivalis* or OMV treatment was associated with increased expression of *Irg-1*. We are currently addressing the impact of itaconate on the mitochondrial remodeling observed in response to *P. gingivalis*. Besides its involvement in regulating macrophage metabolism, itaconate has antibacterial properties (Lampropoulou et al., 2016) and we are also addressing its impact on *P. gingivalis* survival.

The switch to glycolysis from OXPHOS in macrophages treated with *P. gingivalis* and OMVs was associated with significant reprogramming of their metabolic gene expression profile. These changes were broadly consistent to changes apparent in LPS-activated or M1 macrophages (McGettrick and O'Neill, 2013; Jha et al., 2015) and were exemplified by increased expression of key glycolytic genes (e.g., *Glut-1, Hk1/2, Pfkfb3*, and *Pkfl*) and decreased expression of TCA cycle genes (e.g., *Fh1, Pck2*, and *Suclg2*) following *P. gingivalis* or OMV treatment. Notably, one of the most highly regulated genes *Idh1*, which encodes a key enzyme of the TCA cycle and is downregulated in M1 cells leading to mitochondrial dysfunction (Jha et al., 2015), was decreased by OMVs. Indeed, decreased *Idh1* and increased *Irg-1* expression (discussed above) are transcriptional hallmarks of macrophages that have converted to glycolysis (Jha et al., 2015). Not all genes involved in either the glycolytic or TCA pathway were regulated as expected, for example, *Gapdh, Galm*, and *Idh3a*. As a gene involved in the glycolytic pathway *Gapdh* expression was significantly decreased by *P. gingivalis* and its OMVs. Intriguingly, GAPDH functions as a moonlighting protein with diverse functions (O'Neill et al., 2016), which include acting as a surface receptor on macrophages involved in iron acquisition (Raje et al., 2007). It is intriguing to speculate that following infection, *P. gingivalis* downregulates macrophage *Gapdh* expression, in an attempt to impair the removal of extracellular iron, which is an essential nutrient for the growth of *P. gingivalis* and the wider oral biofilm (Lewis, 2010). Flux through the pentose phosphate pathway is typically enhanced in glycolytic macrophages (Haschemi et al., 2012; Jha et al., 2015). Contrastingly, we found that expression of genes in this pathway tended to be decreased with several genes (i.e., *Prps2, Taldo1*, and *Tkt)* being significantly downregulated by *P. gingivalis* or OMVs. The pentose phosphate pathway generates nucleotides and is also a potential source of NADPH for generation of NO and ROS (Haschemi et al., 2012). Whether *P. gingivalis* targets this pathway for protection against macrophage bactericidal activity to contribute to its persistence is unknown. Collectively the metabolic gene expression changes observed in macrophages in response to *P. gingivalis* and OMVs fit with their commitment to ATP generation via glycolysis and the disruption of mitochondrial OXPHOS.

Inflammasomes are cytosolic signaling complexes that specialize in the recognition of a multitude of microbial signals resulting in the generation of the active forms of caspase-1 and IL-1β, which may trigger pyroptotic cell death (Franchi et al., 2012). Pyroptosis allows the elimination of the intracellular niche of pathogens that infect macrophages (e.g., *Shigella, Salmonella)* and exposes them to antimicrobial effector functions (Schaale et al., 2016). Inflammasome activation is distinct in human monocytes compared to macrophages (Netea et al., 2009). In this important study, human monocytes (and PMA-treated THP-1 human monocytic cells) released mature IL-1β after a single stimulation with TLR2 or TLR4 ligands, due to endogenous release of ATP and activation of P2X_7_R. Macrophages on the other hand, are unable to process and secrete IL-1β solely in response to TLR ligands and do not release ATP. Thus, macrophages require a second signal (e.g., exogenous ATP) whereas monocytes (and THP-1 cells) only require one signal for inflammasome activation (Netea et al., 2009). This distinction may neatly explain why *P. gingivalis* activation of the inflammasome and IL-1β production has been demonstrated in THP-1 (Taxman et al., 2006; Park et al., 2014; Jung et al., 2015) and Mono-Mac-6 cell lines (Bostanci et al., 2009; Hamedi et al., 2009), as well as in human monocytes (Huang et al., 2009; Jung et al., 2015). In comparison, studies in mature macrophage populations found that *P. gingivalis* fails to activate the inflammasome (Taxman et al., 2012; Slocum et al., 2014) unless stimulated with a secondary signal (Morandini et al., 2014; Ramos-Junior et al., 2015). These findings are consistent with our own in human and mouse macrophages where we demonstrate that *P. gingivalis* provides the “priming” signal (i.e., upregulation of NLRP3 and pro-IL-1β) but not the secondary triggering signal that leads to maturation of caspase-1 and IL-1β. Thus the reported differences for *P. gingivalis* inflammasome activation are largely due to the nature of the cell type under investigation (i.e., immature monocyte or monocyte cell line vs. mature primary macrophage). Similar to macrophages, gingival epithelial cells do not activate the inflammasome in response to *P. gingivalis* infection and require a second stimulus in the form of exogenous ATP (Yilmaz et al., 2010). Subsequent studies in these cells revealed that *P. gingivalis* possesses a nucleoside-diphosphate kinase (NDK) that inhibits ATP (Johnson et al., 2015) and ROS (Choi et al., 2013; Hung et al., 2013) mediated inflammasome activation (Yilmaz and Lee, 2015). Interestingly, NDK can be secreted from infected cells (Choi et al., 2013) and utilizes the PNX1 membrane hemichannel to be translocated outside of the host cells (Atanasova et al., 2016). Whether similar mechanism(s) operate in macrophages is unknown but together these studies reveal the evasive strategies that enable *P. gingivalis* to circumvent inflammasome activation and host cell death in order to provide an intracellular niche for its survival. It should be noted that other periodontal pathogens, most notably *Aggregatibacter actinomycetemcomitans*, which possesses a potent leukotoxin, can also induce considerable inflammasome activation and pyroptosis. Uniquely, this pathogen's leukotoxin provides both the priming and triggering signals necessary to induce pyroptosis in macrophages (Kelk et al., 2011).

*P. gingivalis* OMVs were also able to provide the necessary priming and triggering signals to potently activate the inflammasome and induce pyroptosis in macrophages. A potential explanation for this finding is that gingipain levels are ~three to five-fold higher on OMVs compared to their parent cells (Mantri et al., 2015), and their proteolytic activity has been found to promote inflammasome activation and cell death responses in different cell types (Sheets et al., 2006; Jung et al., 2015). An additional explanation may be that OMVs do not contain NDK (Veith et al., 2014), so unlike their parent cells, OMVs cannot rely on the NDK inhibition of ATP/ROS-mediated inflammasome activation (Yilmaz and Lee, 2015). Consistent with a role for gingipain activity, we observed that when OMVs were heat-inactivated (which may deactivate gingipain proteolytic function; Stathopoulou et al., 2009) inflammasome and pyroptotic cell death responses in macrophages were diminished. Interestingly, *P. gingivalis* downregulates the production of the gingipains once it resides within the intracellular niche (Xia et al., 2007), which is a strategy not available to OMVs. Such a strategy would presumably lessen the damage to host proteins and reduce inflammasome activation contributing to the pathogen's survival within the host cell. *P. gingivalis* is a highly invasive intracellular oral pathogen that can persist in macrophages (Wang et al., 2007) and endothelial cells (Belanger et al., 2006), and can reside in gingival epithelial cells for extended periods without causing host cell death (Lamont et al., 1995; Lamont and Jenkinson, 1998). *P. gingivalis* is thought to be confined to the autophagosome in macrophages (Wang and Hajishengallis, 2008) whereas bacterial OMVs deliver LPS to the cytosol resulting in inflammasome activation and pyroptosis in macrophages (Vanaja et al., 2016). Thus, it is likely that OMV enrichment for gingipains, coupled to their ability to reach the cytosolic inflammasome complex, explain their marked ability to activate pyroptotic cell death in macrophages. Pyroptotic cell death induced by *P. gingivalis* OMVs (and by other pathogens e.g., *A. actinomycetemcomitans*) may partly explain the increased levels of LDH in the saliva of patients with periodontitis (De La Pena et al., 2007).

This study demonstrates that the major periodontal pathogen *P. gingivalis* promotes a metabolic shift toward glycolysis and disrupts mitochondrial function and the TCA cycle in macrophages. These changes were coupled to remodeling at the transcriptional level with a distinct downregulation of TCA cycle genes and increased glycolytic gene expression consistent with a commitment to ATP generation via aerobic glycolysis. These profound changes were also observed in macrophages in response to *P. gingivalis* OMVs. Uniquely, OMVs triggered considerable inflammatory cytokine release, inflammasome activation and pyroptotic cell death in macrophages. As mentioned (O'Brien-Simpson et al., 2009; Gui et al., 2016), OMVs can penetrate gingival tissue causing tissue damage and inflammation. Our study highlights that this may include activation of inflammatory cytokine release and promotion of glycolysis in gingival tissue macrophage populations leading to their programmed cell death via pyroptosis. The resulting inflammation and release of cytoplasmic compounds [e.g., damage-associated molecular pattern (DAMPs)] into the extracellular milieu would perpetuate local inflammation and, via the gingival exudate flow, return these micronutrients to *P. gingivalis* and other plaque bacteria (Gui et al., 2016). Further investigation of the mechanism(s) regulating this metabolic shift and its contribution to macrophage effector function in response to *P. gingivalis* is warranted.

## Author contributions

AF, ML, WS, AA, and MCL performed the experiments. AF, ML, AC, AM, SD, ER, and JH analyzed and interpreted the data. ML, WS, AC, AM, SD, NO, and ER contributed reagents, materials, or analysis tools. AF and JH drafted the manuscript with all the authors provided the opportunity to comment, conceived and designed the study.

### Conflict of interest statement

The authors declare that the research was conducted in the absence of any commercial or financial relationships that could be construed as a potential conflict of interest.
