# Supplementary figures and images for: Metabolic Remodeling, Inflammasome Activation, and Pyroptosis in Macrophages Stimulated by Porphyromonas gingivalis and Its Outer Membrane Vesicles
Source: Front Cell Infect Microbiol. 2017 Aug 4;7:351. doi: 10.3389/fcimb.2017.00351 (PMC5543041; doi:10.3389/fcimb.2017.00351)

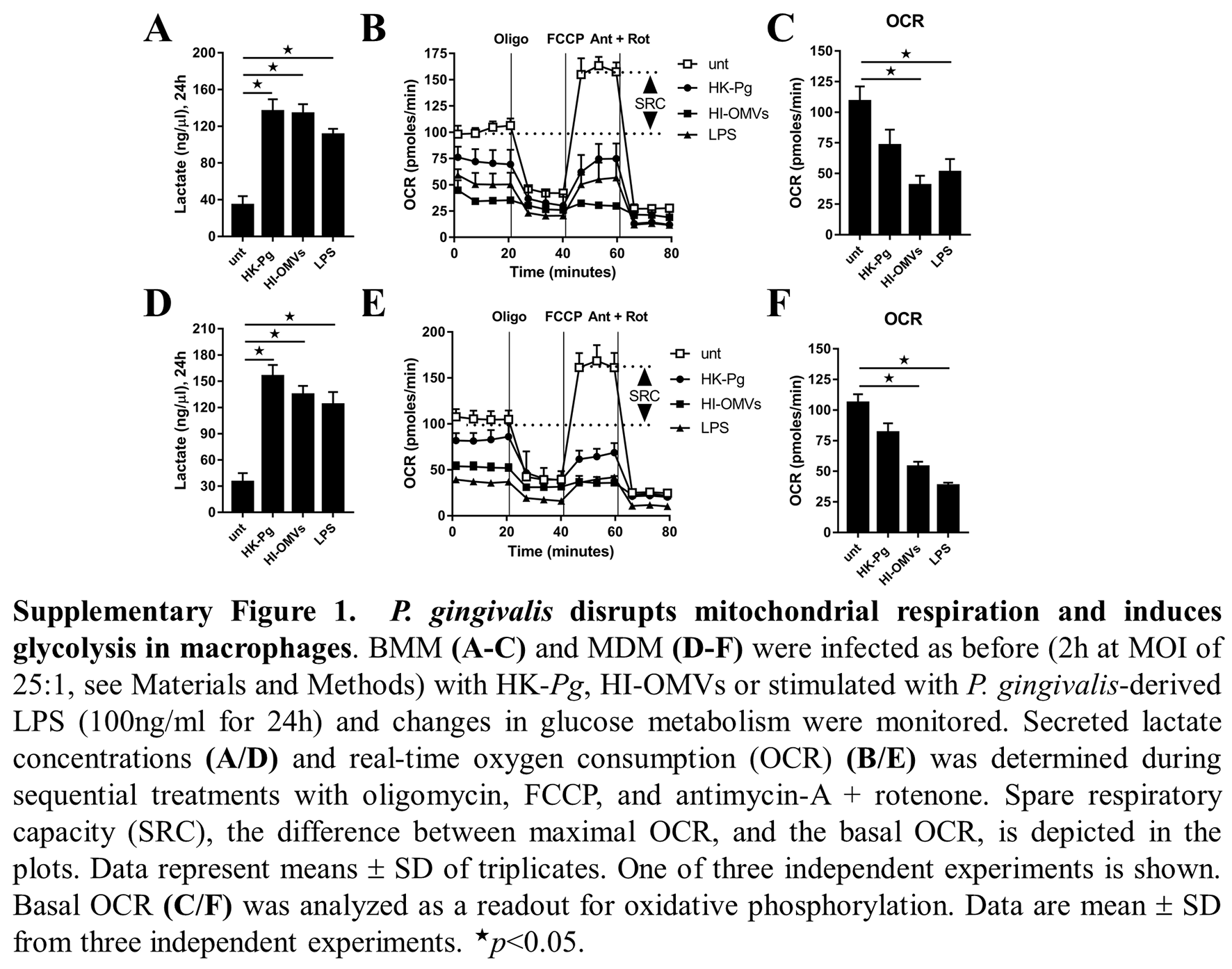

Supplement: Supplementary file 1 [file Image1.tif]

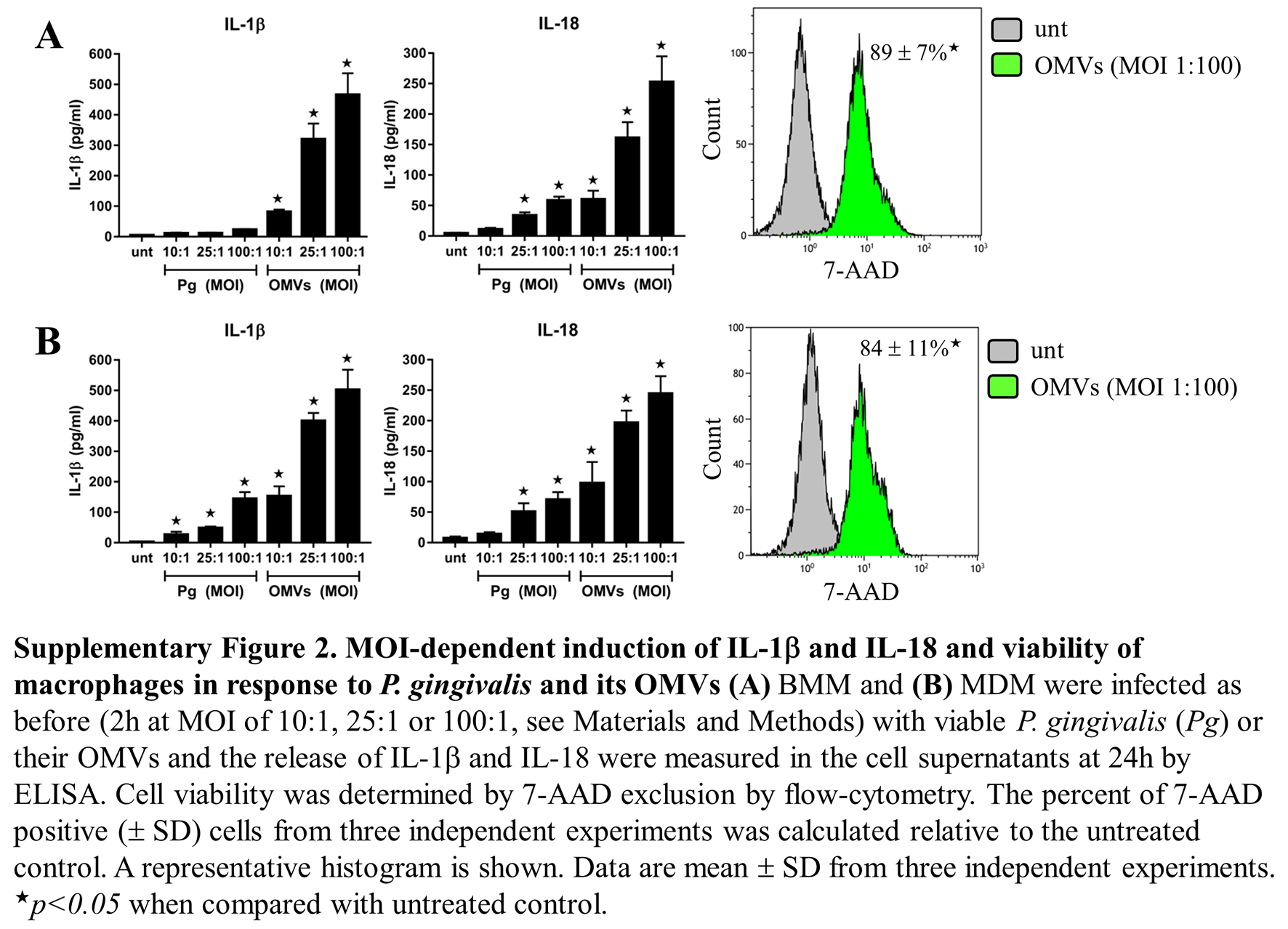

Supplement: Supplementary file 2 [file Image2.TIF]
